# Gatifloxacin Versus Ofloxacin for the Treatment of Uncomplicated Enteric Fever in Nepal: An Open-Label, Randomized, Controlled Trial

**DOI:** 10.1371/journal.pntd.0002523

**Published:** 2013-10-31

**Authors:** Samir Koirala, Buddha Basnyat, Amit Arjyal, Olita Shilpakar, Kabina Shrestha, Rishav Shrestha, Upendra Man Shrestha, Krishna Agrawal, Kanika Deshpande Koirala, Sudeep Dhoj Thapa, Abhilasha Karkey, Sabina Dongol, Abhishek Giri, Mila Shakya, Kamal Raj Pathak, James Campbell, Stephen Baker, Jeremy Farrar, Marcel Wolbers, Christiane Dolecek

**Affiliations:** 1 Oxford University Clinical Research Unit–Nepal, Patan Academy of Health Sciences, Patan Hospital, Patan, Nepal; 2 The Hospital for Tropical Disease, Wellcome Trust Major Overseas Programme, Oxford University Clinical Research Unit–Vietnam, Ho Chi Minh City, Vietnam; 3 Centre for Tropical Medicine, University of Oxford, Oxford, United Kingdom; 4 London School of Hygiene and Tropical Medicine, London, United Kingdom; Massachusetts General Hospital, United States of America

## Abstract

**Background:**

Fluoroquinolones are the most commonly used group of antimicrobials for the treatment of enteric fever, but no direct comparison between two fluoroquinolones has been performed in a large randomised trial. An open-label randomized trial was conducted to investigate whether gatifloxacin is more effective than ofloxacin in the treatment of uncomplicated enteric fever caused by nalidixic acid-resistant *Salmonella enterica* serovars Typhi and Paratyphi A.

**Methodology and Principal Findings:**

Adults and children clinically diagnosed with uncomplicated enteric fever were enrolled in the study to receive gatifloxacin (10 mg/kg/day) in a single dose or ofloxacin (20 mg/kg/day) in two divided doses for 7 days. Patients were followed for six months. The primary outcome was treatment failure in patients infected with nalidixic acid resistant isolates. 627 patients with a median age of 17 (IQR 9–23) years were randomised. Of the 218 patients with culture confirmed enteric fever, 170 patients were infected with nalidixic acid-resistant isolates. In the ofloxacin group, 6 out of 83 patients had treatment failure compared to 5 out of 87 in the gatifloxacin group (hazard ratio [HR] of time to failure 0.81, 95% CI 0.25 to 2.65, p = 0.73). The median time to fever clearance was 4.70 days (IQR 2.98–5.90) in the ofloxacin group versus 3.31 days (IQR 2.29–4.75) in the gatifloxacin group (HR = 1.59, 95% CI 1.16 to 2.18, p = 0.004). The results in all blood culture-confirmed patients and all randomized patients were comparable.

**Conclusion:**

Gatifloxacin was not superior to ofloxacin in preventing failure, but use of gatifloxacin did result in more prompt fever clearance time compared to ofloxacin. Trial registration: ISRCTN 63006567 (www.controlled-trials.com).

## Author Summary

Enteric fever, which comprises of typhoid and paratyphoid fevers, is common in many developing countries. It is also sometimes seen in the Western world in returning travellers. This present study of uncomplicated enteric fever in an outpatient setting in a hospital in Kathmandu, Nepal compared the newer gatifloxacin with the widely-used ofloxacin (two drugs of the fluroquinolone class) in the treatment of this illness. Although fluroquinolones are commonly considered the main group of drugs in the treatment of enteric fever, there have not been comparisons of efficacy between two drugs in this same class in the treatment of enteric fever. Furthermore, certain strains of enteric fever organism called nalidixic-acid resistant strains are proving very difficult to treat in both the local population and the Western travellers. The study focused primarily on the efficacy of the 2 drugs against these particular strains. The results revealed that both drugs were effective but gatifloxacin decreased the patient's fever more rapidly than ofloxacin. Dysglycemia was noted in a 35-year-old woman taking gatifloxacin who did not disclose a pre-existing diagnosis of diabetes at time of enrollment, but not in any other healthy child or young adult.

## Introduction

Enteric fever is endemic in Nepal and many other developing countries [1], [2], [3]. In industrialised countries, it is usually a disease imported by returning travellers [4], most frequently from South Asia [5]. Enteric fever is a systemic infection caused by *Salmonella enterica* serovars Typhi (*S.* Typhi) and Paratyphi A (*S*. Paratyphi A) [6]. For the treatment of uncomplicated enteric fever, the WHO recommends fluoroquinolones for fully sensitive and multidrug resistant (MDR, resistance to chloramphenicol, ampicillin and trimethoprim-sulfamethoxazole) isolates [7]. However, the widespread use of fluoroquinolones for enteric fever has been followed by the emergence of *S.* Typhi *and S.* Paratyphi A isolates with reduced susceptibility to ciprofloxacin (minimum inhibitory concentration (MIC)≥0.125 µg/mL) and ofloxacin (MIC≥0.25 µg/mL) across Asia [8], [9] and parts of Africa [10], [11]. These strains can be identified by high level resistance to nalidixic acid and are associated with specific point mutations in *gyrA* (DNA gyrase) gene, and occasionally the *parC* (topoisomerase IV) gene [12], [13] [8]. Despite these findings, ofloxacin continues to be the standard of care in health facilities in many parts of South and Southeast Asia for the treatment of uncomplicated enteric fever [14], [15], [16].

Gatifloxacin is an 8-methoxyfluoroquinolone which targets both GyrA and topoisomerase IV and hence is less inhibited by the common mutations of the gyrA gene of S typhi than are ciprofloxacin and ofloxacin. [17]. In addition, gatifloxacin had the lowest MICs against nalidixic acid-resistant strains of *S.* Typhi and *S*. Paratyphi A in comparison to other fluroquionolones [8].

In randomized controlled trials (RCTs) carried out in Nepal and Vietnam, gatifloxacin has been shown to be very effective, safe and inexpensive for the treatment of enteric fever [18],[19],[20].

Although WHO recommends fluoroquinolones for the treatment of enteric fever, a direct comparison between two fluoroquinolones in a large randomized trial designed with clinically relevant endpoints has not been performed. The most recent Cochrane review remarks, “There is some evidence that the newest fluoroquinolone, gatifloxacin, remains effective in some regions where resistance to older fluoroquinolones has developed. However, the different fluoroquinolones have not been compared directly in trials in these settings” [15]. We therefore chose to compare ofloxacin because of its widespread use in the treatment of enteric fever with the newer gatifloxacin.

The objective of this trial was to conduct an open label, randomised clinical comparison of gatifloxacin versus ofloxacin for the treatment of uncomplicated enteric fever in an area with a high proportion of nalidixic acid-resistant isolates. This trial was performed in an outpatient setting, reflecting the “real life situation” in resource-poor countries where enteric fever is endemic.

## Methods

### Ethics

The trial was approved by the Nepal Health Research Council, Kathmandu, Nepal and the Oxford Tropical Research Ethics Committee, Oxford, UK and was conducted according to the principles of the declaration of Helsinki. The trial was registered as ISRCTN63006567 (www.controlled-trials.com). The Independent Data and Safety Monitoring Board (DSMB) provided oversight of the study and reviewed the data from the first 50 patients with blood culture-confirmed enteric fever in each treatment group. A full written informed consent was obtained from all the study participants [18], [20]. Written informed consent was obtained by the parent or guardian of participating children (under 18 years of age).

### Patients

Patients with fever for more than three days who were clinically diagnosed to have enteric fever (undifferentiated fever with no clear focus of infection on preliminary physical examination and laboratory tests), presenting to the outpatient or emergency department of Patan Hospital, Lalitpur, Nepal from July 2008 to August 2011, whose residence was in a designated area of 20 km^2^ in urban Lalitpur and who gave fully informed written consent were eligible for the study [18], [20].

Exclusion criteria were pregnancy or lactation, age under 2 years or weight less than 10 kg, shock, jaundice, gastrointestinal bleeding or any other signs of severe enteric fever, previous history of hypersensitivity to either of the trial drugs, or known previous treatment with chloramphenicol, fluoroquinolones, third generation cephalosporins, or macrolides within one week of hospital admission. Patients pretreated with amoxicillin or cotrimoxazole were included as long as they did not show evidence of clinical response.

### Randomisation and masking

Randomisation was performed in blocks of 50 without stratification by a clinical trial administrator who was not involved in the study. Random allocations were placed in sealed opaque envelopes, which were kept in a locked drawer and opened by trained community medical auxiliaries (CMAs) who were responsible to administer the drugs, once each patient was enrolled into the trial after meeting the inclusion and exclusion criteria and giving written consent. The treating study physicians were blinded throughout the study regarding the treatment allocation. Patients were enrolled in the order they presented and the sealed envelopes were opened in strict numerical sequence. Masking was not possible because of the differing drug intake schedule.

### Procedures

Each enrolled patient was randomly assigned to treatment with either gatifloxacin (400 mg tablets, Square Pharmaceutical Limited, Bangladesh) at 10 mg per kg per day in a single oral dose for 7 days or ofloxacin (200 mg or 400 mg tablets, National Healthcare Pvt. Ltd., Nepal) at 20 mg per kg per day in two divided oral doses for 7 days. Gatifloxacin and ofloxacin tablets were cut and weighed and the patients' daily doses were prepared in sealed plastic bags. For example, for the gatifloxacin arm, each patient was given doses nearest to 10 mg/kg for that particular patient erring on the higher side but not exceeding by 10 mg.

After enrolment, patients were managed as outpatients as described previously [20], [18]. The CMAs made a visit to each patient's house twice a day (morning and evening) for 10 days or until the patient was afebrile and without symptoms. The intake of each dose of ofloxacin or gatifloxacin was directly observed by the CMAs. The physicians re-examined the patients on days 8 and 15 and at 1, 3, and 6 months. All examinations were standardized and entered on case record forms.

Complete blood counts were performed on days 1 and 8. On day 1, serum creatinine, bilirubin, aspartate aminotransferase (AST) and alanine aminotransferase (ALT) were measured. Random plasma glucose was measured on day 1, day 8, day 15 and 1 month. On days 2 to 7, during the evening home visit, blood glucose was measured by finger-prick testing (One Touch Sure Step, Johnson and Johnson, USA) by the CMAs. Heamoglobin A1c was measured at 3 months.

Three (for children under 12 years) or seven mL (for those above 12 years) of blood were collected for microbiological blood culture from all patients at enrolment, from culture positive patients on day 8, and if symptoms suggested a clinical relapse. Blood samples were inoculated into media containing tryptone soya broth and sodium polyanethol sulphonate, up to a total volume of 50 mL. The bottles were incubated at 37°C and examined daily for bacterial growth over seven days. On observation of turbidity, media was sub-cultured onto MacConkey agar plate to isolate *Salmonella* serotypes. Isolates were screened using standard biochemical tests and *S. Typhi* and *S. Paratyphi* A were identified using AP120E (Bio Merieux, Paris, France) and slide agglutinaton with specific antisera (Murex Biotech, Dartford, UK).

Stool cultures were performed on day 1 in all patients, in blood culture-positive patients after completion of treatment and at the 1, 3 and 6 months visits. Stool specimens were inoculated into 10 mL of Selenite F broth and incubated at 37°C. After the overnight incubation, the broth was subcultured onto MacConkey agar and xylose lysine decarboxylase agar media.

MICs of nalidixic acid, ofloxacin, ciprofloxacin, gatifloxacin, azithromycin, chloramphenicol, ampicillin and ceftriaxone were determined by E-test (AB Biodisk, Solna, Sweden) according to the manufacturer's instructions.

### Outcomes

The primary endpoint of this study was the composite endpoint of treatment failure, which was defined by the occurrence of any of the following: persistence of fever of more than 37.5°C at day 10 of treatment; need for rescue treatment with ceftriaxone or azithromycin as judged by the treating physician; microbiological failure, defined as a positive blood culture for *S.* Typhi or *S.* Paratyphi A on day 8; relapse, defined as the reappearance of symptoms of enteric fever between day 8 to day 31 in patients who were initially categorized as successfully treated, this included culture-confirmed (including mismatch of serotypes [e.g., day 1 blood culture positive for *S.* Typhi and relapse blood culture positive for *S.* Paratyphi A or vice versa]) and syndromic enteric fever, and occurrence of enteric fever related complications. Time to treatment failure was defined as the time from the first dose of treatment until the date of the earliest failure event of that patient, and patients without an event were censored at the date of their last follow-up visit.

Secondary endpoints were: fever clearance time (FCT, time from the first dose of treatment given until the temperature is for the first time ≤37.5°C and the patient remained afebrile for at least 48 hours); time to relapse until day 31, day 62, or 6 months of follow-up; and faecal carriage at the follow-up visits at 1, 3 and 6 months. The patients' FCTs were calculated electronically on the basis of twice-daily recorded temperatures. Patients without recorded FCT or relapse were censored at the date of their last follow-up visit. To reduce possible bias, an investigator who was not involved in the recruitment of patients decided patients' final outcomes by use of a masked database.

The analysis plan also predefined a modified secondary definition (“modified analysis”) of the primary endpoint of this study, treatment failure (see above), in which persistent fever of more than 37.5°C at day 7 replaced persistent fever on day 10 as part of the composite endpoint. This was done to allow comparison with previous studies [21] and to explore the modification of this endpoint for future definitions of outcomes for standardisation of clinical trials in enteric fever.

### Statistical analysis

The trial was powered as a superiority trial to detect a 20% decrease in the risk of treatment failure due to gatifloxacin (from 25% for ofloxacin to 5% for gatifloxacin) in the treatment of enteric fever patients infected with nalidixic acid-resistant isolates. To achieve 90% power at the two-sided 5% significance level, 75 patients per group would be required and the original protocol specified a sample size of 100 patients with culture-confirmed enteric fever in each arm. A blinded interim observation was performed after 510 patients had been recruited and based on this, the study team decided to amend the protocol and increase the sample size to obtain 110 blood culture positive patients in each arm so that at least 75 patients with nalidixic acid-resistant *S*. Typhi or *S.* Paratyphi A in each arm could be followed up for one month and analysed. On the basis of results from a previous study [18], we assumed that approximately 40% of recruited patients had culture-confirmed enteric fever. To allow for some loss to follow-up, a total of 629 patients with suspected enteric fever were recruited to the trial.

The times to treatment failure, fever clearance, and relapse, were summarised by Kaplan-Meier estimates and compared between interventions using Cox regression models with the treatment group as the only covariate. For the primary endpoint (treatment failure), we also compared the absolute risk of treatment failure until day 31 based on Kaplan-Meier estimates and standard errors according to Greenwood's formula [22]. Additionally, the times to treatment failure and FCT were analysed in the subgroups defined by culture result, pathogen (*S.* Typhi or *S.* Paratyphi A), age (<16 years or ≥16 years), MICs of gatifloxacin and ofloxacin, and heterogeneity of the treatment effect was tested with Cox regression models that included an interaction between treatment and subgroup.

The primary analysis population was the population infected with nalidixic acid-resistant isolates (a subgroup of the population with blood culture-confirmed enteric fever). Statistical analyses were also performed for all blood culture positive patients (blood culture positive population) and all patients who were assigned treatment, with the exception of those patients who were mistakenly randomised or withdrew before the first dose of study treatment (intention to treat population, ITT, this included patients with negative blood culture result) for treatment failure and safety. All reported tests were done at the two-sided 5% significance level, and 95% CIs are reported. All analyses were performed with the statistical software R version 2.15.1 [23].

## Results

**Figure 1:**
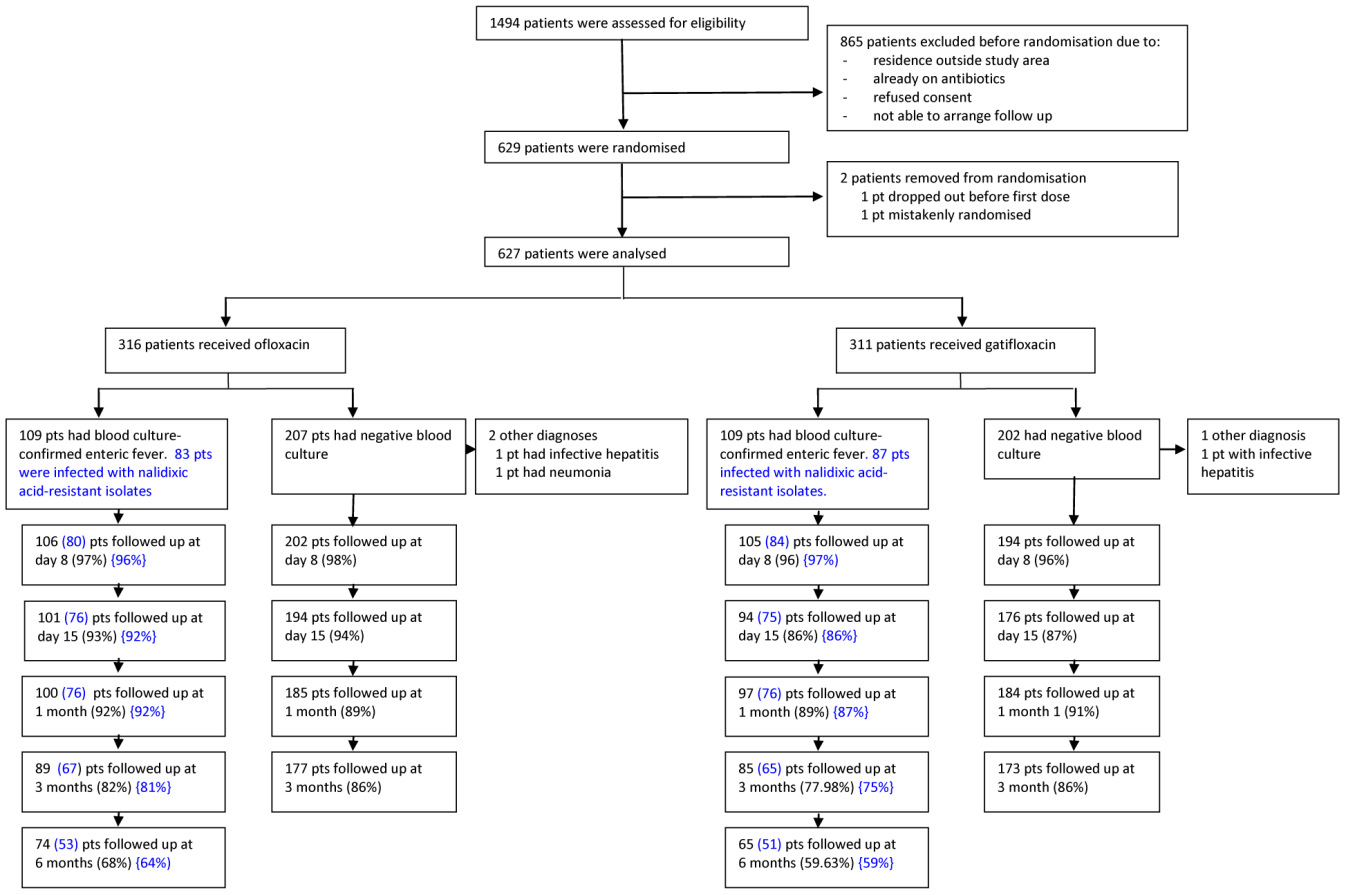
Trial profile. Numbers in blue font represent numbers of patients infected with nalidixic acid resistant isolates.

The study flow is displayed in Figure 1. Of the 1494 patients who were assessed for eligibility, 865 were excluded prior to randomisation, primarily due to residence outside the designated study area. Two randomised patients were excluded from all analyses (Figure 1), leaving 627 patients in the intention to treat population (ITT). Table 1 shows the baseline characteristics of these patients. Only 2 patients in the ofloxacin arm and 4 patients in the gatifloxacin arm had a positive stool culture for *S.* Typhi or *S*. Paratyphi A before the start of treatment (Table 1).

**Table 1:** Baseline characteristics of patients according to treatment group (intention to treat population).

	Ofloxacin group (n = 316)	Gatifloxacin group (n = 311)
Age in years, median (IQR)	16 (9–24)	17 (10–22)
Male sex	199 (63%)	207 (67%)
Weight in kg, median (IQR)	45 (22–53)	45 (26–54)
Received Amoxicillin	36(11%)	28(9%)
Duration of illness before admission in days, median (IQR)	5 (4–7)	5 (4–7)
Temperature at admission in °C, median (IQR)	38.6 (38.2–39.0)	38.7 (38.1–39.2)
Headache, number (%)	272 (87%)	267 (86%)
Anorexia, number (%)	230 (74%)	222 (72%)
Abdominal pain, number (%)	132 (42%)	122 (40%)
Cough, number (%)	113 (36%)	128 (41%)
Nausea, number (%)	80 (26%)	89 (29%)
Vomiting, number (%)	64 (20%)	50 (16%)
Diarrhoea, number (%)	52 (17%)	49 (16%)
Constipation, number (%)	34 (11%)	40 (13%)
Hepatomegaly, number (%)	2 (1%)	5 (2%)
Splenomegaly, number (%)	4 (1%)	2 (1%)
Haematocrit in %, median (IQR)	38 (36–42)	39 (36–42)
Leucocyte count {×10^9^ cells/L}, median (IQR)	6.0 (4.7–7.7)	6.1 (4.8–7.7)
Platelet count {×10^9^cells/L}, median (IQR)	172 (148–216)	175 (142–216)
AST {U/L}, median (IQR)	47 (33–66)	46 (35–68)
ALT {U/L}, median (IQR)	41 (26–64)	44 (28–69)
*Salmonella* Typhi isolated	66	66
*Salmonella* Paratyphi A isolated	43	43
Positive pretreatment faecal cultures	2 (0.78%)	4 (1.52%)

AST = serum aspartate aminotransferase (normal range 12–30 U/L), ALT = serum alanine aminotransferase (normal range 13–40 U/L).

**Figure 2:**
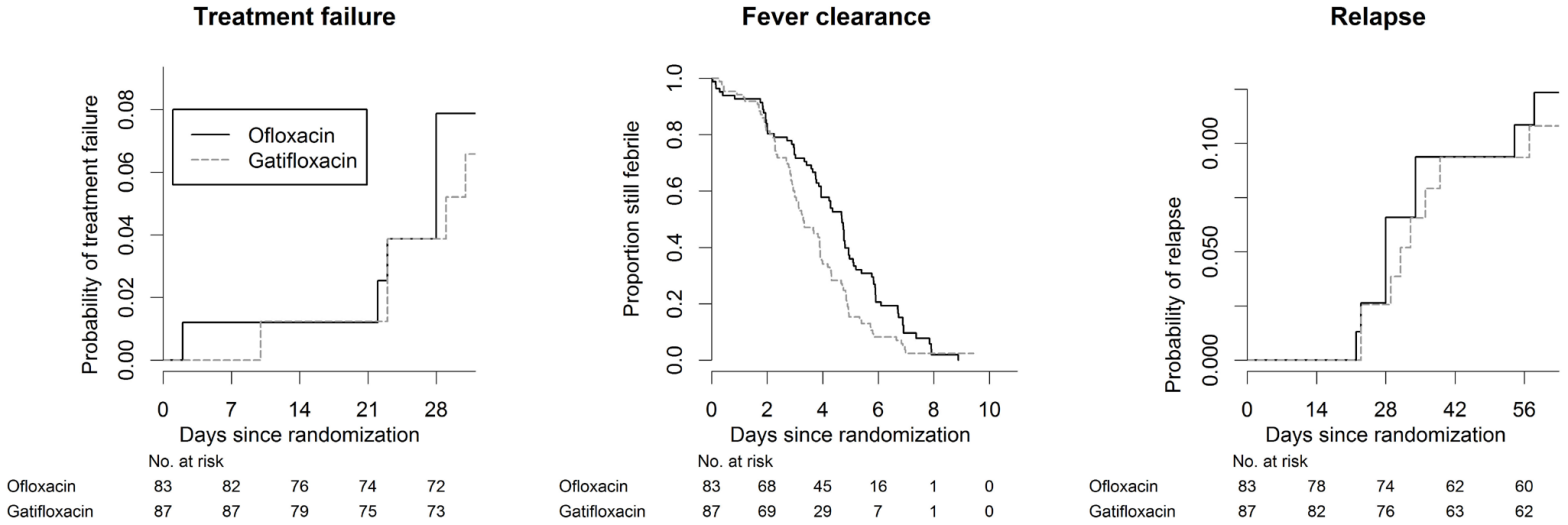
Kaplan-Meier estimates for patients infected with nalidixic acid resistant isolates. Kaplan- Meier estimates of the probability of treatment failure, fever clearance time, and the probability of relapse for patients infected with nalidixic acid resistant isolates.

The outcomes for the primary analysis population, the 170 patients infected with nalidixic acid-resistant isolates, are summarised in Table 2 and Figure 2. The number of patients with treatment failure was 6/83 in the ofloxacin group and 5/87 in the gatifloxacin group (Hazard Ratio, HR = 0.81, 95% CI 0.25 to 2.65; p = 0.73). One patient in the gatifloxacin arm had persistent fever on day 10 and received azithromycin treatment (1 g per day) starting on day 11. There were 9 relapses (Table 2) within 31 days after the start of treatment (5 in the ofloxacin group and 4 in the gatifloxacin group) and all nine patients responded well to azithromycin (20 mg/kg, up to 1 g per day) for 7 days. One patient in the ofloxacin group had severe abdominal pain on day 2 and was admitted to hospital with the presumed diagnosis of appendicitis. He stayed in hospital overnight for observation and azithromycin treatment was started. The next day, the patient had improved significantly and went home. This was the only patient in this trial who had possible enteric fever related complications (Table 2) and needed hospitalization.

**Table 2:** Summary of primary and secondary endpoints for patients infected with nalidixic acid-resistant isolates of *S.* Typhi and *S*. Paratyphi A (primary analysis population).

	Ofloxacin group (n = 83)	Gatifloxacin group(n = 87)	Comparison
**Time to treatment failure** #			
Total number of pt with failures$	6	5	HR = 0.81 (CI 0.25 to 2.65), p = 0.73
- Persistent fever on day 10	0	1	
- Need for rescue treatment	1	0	
- Microbiological failure	0	0	
- Relapse until day 31	5	4	
- Enteric fever related complications	1	0	
**Risk of treatment failure** 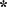	0.08 (CI 0.02 to 0.14)	0.07 (CI 0.01 to 0.12)	RD = −0.01 (CI −0.10 to 0.07), p = 0.76
**Median (IQR) time to fever clearance (days)** 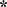	4.70 (2.98 to 5.90)	3.31 (2.29 to 4.75)	HR = 1.59 (CI 1.16 to 2.18), p = 0.004
**Relapses until day 31 – n**	5	4	HR = 0.77 (CI 0.21 to 2.87); p = 0.70
- n blood culture-confirmed	3	2	
- n syndromic	2	2	
- Probability of relapse 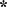	0.07 (CI 0.01 to 0.12)	0.05 (CI 0.001 to 0.10)	
**Relapses until day 62 – n**	9	8	HR = 0.86 (CI 0.33 to 2.23); p = 0.75
- n blood culture-confirmed	4	3	
- n syndromic	5	5	
- Proportion 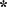	0.12 (CI 0.04 to 0.20)	0.11 (CI 0.03 to 0.18)	
**Relapses after day 62 – n**	2	2	-
- n blood culture-confirmed	0	0	
- n syndromic	2	2	

$ Patients may have more than one type of treatment failure.

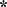
Kaplan-Meier estimates.

HR = Hazard ratio (based on Cox regression), RD = absolute risk difference (based on Kaplan-Meier estimates), CI = 95% confidence interval interval, IQR = inter-quartile range.

n number of patients; pt patients.

#Footnote: If persistent fever on day 7 (instead of day 10) would already be considered a treatment failure event (“modified analysis”), then there would be 21 treatment failures in the ofloxacin group *vs*. 11 in the gatifloxacin group (with 16 *vs*. 7 patients with persistent fever on day 7): HR = 0.46 (CI 0.22–0.96), p = 0.04.

There was also no evidence of difference in treatment failure rates between treatment groups amongst all patients with blood culture confirmed enteric fever (Table S1), the ITT population (Table S2) or in any of the predefined subgroups, which were age (less than 16 years or 16 years and above), pathogen (*S.* Typhi or *S*. Paratyphi A) and MIC of the isolates (Table 3).

**Table 3:** Treatment failure – subgroup analyses.

Subgroup	Ofloxacin group		Gatifloxacin group		HR (95% CI), p-value	p-value for heterogeneity 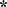
	n	#failures (risk)	n	#failures (risk)		
Intention to treat population	316	13 (0.04)	311	8 (0.03)	0.63 (0.26 to 1.15), p = 0.30	-
Population						0.99
- Culture confirmed pts	109	8 (0.08)	109	5 (0.05)	0.62 (0.20 to 1.90), p = 0.40	
- Culture negative pts	207	5 (0.03)	202	3 (0.02)	0.62 (0.15 to 2.58), p = 0.51	
Nalidixic acid resistance (in culture confirmed pts)						
- MIC≤16 mg/ml (susceptible)	21	0 (0.00)	20	0 (0.00)	- (no events)	-
- MIC≥32 mg/ml (resistant)	83	6 (0.08)	87	5 (0.07)	0.81 (0.25 to 2.65), p = 0.73	
Ofloxacin MIC (in culture confirmed pts)						
- MIC≤0.125 mg/ml (susceptible)	19	0 (0.00)	19	0 (0.00)	- (no events)	-
- MIC between 0.25 and 0.75 mg/ml	61	6 (0.11)	67	5 (0.08)	0.74 (0.23 to 2.43), p = 0.62	
- MIC≥1 mg/ml (resistant)	24	0 (0.00)	21	0 (0.00)	- (no events)	
Gatifloxacin MIC (in culture confirmed pts)						0.05#
- MIC≤0.19 mg/ml	63	6 (0.11)	65	2 (0.03)	0.29 (0.06 to 1.46), p = 0.13	
- MIC≥0.25 mg/ml	41	0 (0.00)	42	3 (0.09)	8.01 (0.78 to 1078), p = 0.08#	
Pathogen (in culture confirmed pts)						0.05
- *Salmonella* Typhi	66	7 (0.12)	66	2 (0.03)	0.26 (0.05 to 1.27), p = 0.10	
- *Salmonella* Paratyphi A	43	1 (0.02)	43	3 (0.08)	3.26 (0.34 to 31.42), p = 0.31	
Age (all patients)						0.83
- Less than 16 years	148	6 (0.04)	134	3 (0.02)	0.56 (0.14 to 2.24), p = 0.41	
- 16 years or older	167	7 (0.05)	174	5 (0.03)	0.68 (0.22 to 2.15), p = 0.51	
Age (in culture confirmed patients)						0.49
- Less than 16 years	48	5 (0.11)	44	2 (0.05)	0.44 (0.08 to 2.25), p = 0.32	
- 16 years or older	60	3 (0.05)	63	3 (0.05)	0.96 (0.19 to 4.75), p = 0.96	

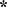
Heterogeneity was tested with a Cox regression model that included an interaction between treatment effect and subgroup.

# Based on Cox regression with Firth's penalized likelihood to cope with separation.

MIC's and age were missing for some patients. N number, pts patients, MIC minimum inhibitory concentration.

In contrast, fever clearance times (FCT) were significantly shorter in the gatifloxacin arm of the study. In patients infected with nalidixic acid-resistant isolates, the median FCT was 4.70 (IQR 2.98 to 5.90) days in the ofloxacin arm and 3.31 (IQR 2.29 to 4.75) days in the gatifloxacin arm (HR = 1.59, 95% CI 1.16 to 2.18; p = 0.004). The comparison also reached statistical significance in patients with blood culture confirmed enteric fever and the ITT population and there was no convincing evidence of heterogeneity in any of the predefined subgroups (Tables S1 and S2, Table 4).

**Table 4:** Fever clearance time – subgroup analyses.

Subgroup	Ofloxacin group		Gatifloxacin group		HR (95% CI), p-value	p-value for heterogeneity 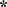
	n	Median FCT (days)	n	Median FCT (days)		
Intention to treat population	316	2.15	311	1.97	1.20 (1.02 to 1.42), p = 0.03	-
Population						0.78
- Culture confirmed pts	109	3.99	109	3.30	1.41 (1.07 to 1.86), p = 0.01	
- Culture negatives	207	1.70	202	1.28	1.16 (0.95 to 1.41), p = 0.16	
Nalidixic acid resistance (in culture confirmed pts)						0.08
- MIC≤16 g/ml (sensitive)	21	2.93	20	2.91	0.83 (0.43 to 1.57), p = 0.56	
- MIC≥32 g/ml (resistant)	83	4.70	87	3.31	1.59 (1.16 to 2.18), p = 0.004	
Ofloxacin MIC (in culture confirmed pts)						0.10
- MIC≤0.125 g/ml (susceptible)	19	2.82	19	2.95	0.73 (0.37 to 1.43), p = 0.36	
- MIC between 0.25 and 0.75 g/ml	61	4.76	67	3.31	1.70 (1.18 to 2.46), p = 0.004	
- MIC≥1 g/ml (resistant)	24	4.24	21	3.44	1.28 (0.69 to 2.37), p = 0.44	
Gatifloxacin MIC (in culture confirmed pts)						0.59
- MIC≤0.19 g/ml	63	3.83	65	3.05	1.51 (1.04 to 2.18), p = 0.03	
- MIC≥0.25 g/ml	41	4.51	42	3.68	1.38 (0.88 to 2.15), p = 0.16	
Pathogen (in culture confirmed pts)						0.67
- *Salmonella* Typhi	66	3.89	66	3.13	1.32 (0.92 to 1.88), p = 0.13	
- *Salmonella* Paratyphi A	43	4.70	43	3.51	1.53 (0.98 to 2.38), p = 0.06	
Age (all patients)						0.38
- Less than 16 years	148	2.00	134	2.10	1.14 (0.90 to 1.45), p = 0.28	
- 16 years or older	167	2.28	174	1.93	1.29 (1.04 to 1.62), p = 0.02	
Age (culture confirmed pts)						0.32
- Less than 16 years	48	3.99	44	3.76	1.29 (0.84 to 1.98), p = 0.24	
- 16 years or older	60	4.28	63	3.24	1.63 (1.12 to 2.38), p = 0.01	

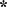
Heterogeneity was tested with a Cox regression model that included an interaction between treatment effect and subgroup.

MIC's and age were missing for some patients. N number, pts patients, MIC minimum inhibitory concentration.

Of note, in the predefined modified analysis that we conducted, in which persistent fever on day 7 replaced persistent fever on day 10 as part of the composite primary endpoint, there was a significant difference in the number of patients with treatment failure in favour of gatifloxacin in all three groups (see also footnotes Tables S1 and S2). Using this seven-day cut off, in the population infected with nalidixic acid-resistant isolates, there were 21 out of 83 patients with treatment failure in the ofloxacin group *versus* 11 patients out of 87 in the gatifloxacin group (HR = 0.46, 95% CI 0.22 to 0.96, p = 0.04; 16 *versus* 7 patients were still febrile on day 7).

During the six months of follow up, only one patient in the blood culture positive group had a positive stool culture. This occurred at the end of one month and the patient was treated with ofloxacin. Only syndromic relapses were documented after day 62 in all three patient populations (Table 2, Tables S1 and S2), with the exception of one culture negative patient (ITT population) in the gatifloxacin group who experienced a culture confirmed relapse on day 92.

Adverse events were analysed in the ITT population. 215 out of 316 (68%) patients in the ofloxacin group and 223 out of 311 (72%) patients in the gatifloxacin group experienced an adverse event (Table S3). Most adverse events were mild (grade 1 or grade 2). Two patients, one from the gatifloxacin and one from the ofloxacin group developed generalized skin rash on day 3, which disappeared after stopping the drugs. Another patient in the gatifloxacin arm had generalized discomfort and treatment was stopped on day 4. He was eventually diagnosed with pulmonary TB. Another patient (described in the outcomes paragraph above) had abdominal pain and was admitted to hospital with the presumptive diagnosis of appendicitis. Finally a patient treated with gatifloxacin had a random blood glucose level of 280 mg/dl on two different occasions (days 3 and 5) and gatifloxacin was stopped on day 5.

In total only five patients, four blood cultures negative and one blood culture positive patient had their treatment discontinued due to presumed adverse events. All of them, except the patient with TB, were started on azithromycin (20 mg/kg up to 1 g per day) for 7 days and improved.

The proportion of patients with haemoglobin A1c levels >6% at the end of three months was similar in both groups (48/262 (18%) patients in the ofloxacin group and 48/248 (19%) patients in the gatifloxacin group).

The MIC results for the 218 available *S.* Typhi and *S*. Paratyphi A isolates are shown in Table 5. Eighty-four out of 86 (97.6%) of the S. Paratyphi A and 86 out of 132 (65.1%) of the *S.* Typhi strains were nalidixic acid resistant. None of the isolates were MDR or demonstrated ceftriaxone resistance. The MIC50s and MIC90s were consistently higher for the *S*. Paratyphi A isolates than for *S.* Typhi isolates.

**Table 5:** Minimum inhibitory concentration MIC of *Salmonella* Typhi and paratyphi A.

	*Salmonella* Paratyphi A (n = 86) 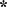	*Salmonella* Typhi (n = 132)*	p value
Nalidixic Acid			
MIC 50 (µg/mL)	>256.00	>256.00	
MIC 90 (µg/mL)	>256.00	>256.00	
Range	>256.00 to >256.00	0.50 to >256.00	<0.0001
Ofloxacin			
MIC 50 (µg/mL)	1.00	0.25	
MIC 90 (µg/mL)	1.50	0.38	
Range	0.38 to 1.50	0.01 to 1.00	<0.0001
Ciprofloxacin			
MIC 50 (µg/mL)	0.38	0.19	
MIC 90 (µg/mL)	0.50	0.38	
Range	0.19 to 0.75	0.00 to 1.50	<0.0001
Gatifloxacin			
MIC 50 (µg/mL)	0.38	0.09	
MIC 90 (µg/mL)	0.38	0.12	
Range	0.12 to 0.50	0.00 to 0.50	<0.0001
Azithromycin			
MIC 50 (µg/mL)	4.00	3.00	
MIC 90 (µg/mL)	6.00	4.00	
Range	1.50 to 6.00	0.50 to 6.00	<0.0001
Chloramphenicol			
MIC 50 (µg/mL)	4.00	4.00	
MIC 90 (µg/mL)	6.00	6.00	
Range	3.00 to 8.00	1.50 to 8.00	<0.0001
Ampicillin			
MIC 50 (µg/mL)	1.00	0.75	
MIC 90 (µg/mL)	1.50	0.85	
Range	0.50 to 3.00	0.25 to 1.00	<0.0001
Nalidixic acid resistant isolates	84 (98%)	86 (65%)	<0.0001

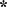
132 *S*.Typhi and 86 *S*.Paratyphi A were available for MIC testing. MIC 50/90 = concentration at which 50% and 90% of the organisms, respectively, are inhibited. Comparison based on Fisher's exact test for categorical data and Wilcoxon test for continuous data.

**Figure 3:**
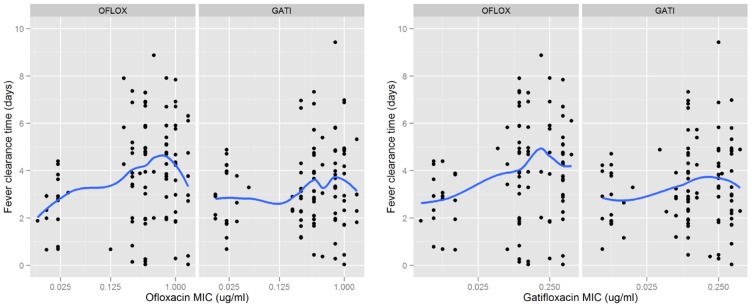
Scatter plots of drugs MIC versus fever clearance time. Gatifloxacin and ofloxacin MICs versus fever clearance time by treatment group for patients with blood culture confirmed enteric fever. Blue lines correspond to LOESS scatter plot smoothers.

Higher (log-transformed) MICs to ofloxacin and gatifloxacin were associated with a prolonged FCT in both study arms: ofloxacin group (p = 0.0003 for ofloxacin MIC; 0.0006 for gatifloxacin MIC) and significant in the gatifloxacin group (p = 0.03 for both ofloxacin MIC and gatifloxacin MIC) (Figure 3).

## Discussion

Gatifloxacin was not superior to ofloxacin in preventing treatment failure. Ofloxacin with adequate dosing (20 mg/kg per day) treated enteric fever caused by nalidixic acid resistant strains successfully, but with longer fever clearance times than gatifloxacin. In the context of the data of this trial, we would like to discuss some key issues in the treatment of enteric fever. The emergence and spread of MDR and nalidixic acid-resistant *S.* Typhi and *S.* Paratyphi A in Asia and parts of Africa has limited the number of effective antimicrobials available for treatment [8],[9], [10]. The other issues relate to the design of clinical trials in enteric fever, especially the definition of efficacy outcomes and the implications of those definitions on the results. We have conducted a series of randomized controlled trials to document the best treatment options for enteric fever caused by nalidixic acid-resistant *S*. Typhi and *S*. Paratyphi *A* [18], [19]. In these trials, gatifloxacin has shown to be an effective and safe treatment for enteric fever. Despite a high proportion of nalidixic acid-resistant isolates, the older generation fluoroquinolone ofloxacin is still used as standard of care in health facilities in South and South East Asia. Clinical trials in Vietnam showed a reduced efficacy of ofloxacin in the treatment of nalidixic acid-resistant enteric fever [21],[24]. Studies have shown that gatifloxacin works against mutated forms of the gyrA and ParC against which the older fluoroquinolones like ciprofloxacin do not work [17]. Although other fluoroquinolones like ciprofloxacin and ofloxacin also target gyrA and ParC, the C-8-methoxy group in gatifloxacin works to inhibit the resistant mutants not inhibited by the older fluoroquinolones [8], [17].

Therefore we hypothesized that gatifloxacin may perform better clinically with lower treatment failure rates than the older fluoroquinolone ofloxacin in a setting where there is a high(80%) proportion of nalidixic acid resistance.

We therefore conducted a direct comparison of ofloxacin and gatifloxacin, and our primary population of interest was the patients infected with nalidixic acid–resistant isolates. The continued use of ofloxacin in Asia is also partially caused by the lack of availability of gatifloxacin. Gatifloxacin has been withdrawn from the US and some other countries in 2006, following a retrospective report of an increased risk of dysglycaemia in elderly Canadian outpatients [25]. In 2011, gatifloxacin was banned in India. Previous case reports have highlighted an effect on glucose homeostasis in patients with non-insulin-dependent diabetes on therapy and elderly patients with age-related decreases in renal function [26]. However, patients with enteric fever are typically children and young adults, who are generally healthy and have good kidney function. Over the last few years, more than 1,123 patients (children and adults) suffering from enteric fever [18],[19],[20];and this trial), 249 children with shigellosis [27] and 15 adult patients with TB meningitis [28] have been treated with gatifloxacin in registered randomised clinical trials in Nepal and Vietnam, and no problems in glucose homeostasis have been observed. In a previous study [18] and in this currently reported trial, as an additional safety measure, random blood glucose was monitored daily for 7 days, at day 15 and one month and HbA1c was measured at 3 months. One patient out of 628 patients who received gatifloxacin and were monitored in these 2 trials showed hyperglycemia. She was a 35 year old woman who did not reveal on enrollment that she was intermittently taking oral hypogylcemic drugs for diabetes. Her fever improved with azithromycin that was started on day 5 and she was followed as an outpatient for diabetes. Gatifloxacin is also under investigation as an alternative drug in short-course tuberculosis regimen. In a multicentre trial in Africa, 917 patients received gatifloxacin daily for four months as part of a drug combination regimen for the treatment of pulmonary tuberculosis.Dysglycemia has not emerged as an adverse event in this population [29].

Clearly, the risk-benefit ratio of gatifloxacin is very different in the two patient populations; on one side, the elderly and multi-morbid Canadian population and on the other side, a young and otherwise healthy population suffering from infectious diseases, like enteric fever and tuberculosis. To conduct these trials, we have considered very carefully the design of clinical trials in enteric fever. Previous Cochrane reviews have criticised the small number of patients enrolled and the varying methodological quality of enteric fever trials [15], [16]. To address the technical issue of the low sensitivity of microbiological blood culture (an estimated 40 to 60% of clinical suspected enteric fever), we have included the culture negative population in all analyses and added symptomatic relapse (not confirmed by culture) to the outcome events of all our studies. We used a composite endpoint, treatment failure, evaluated at 1 month, which included the following unfavourable events: persistent fever at day 10, need of rescue treatment, positive blood culture for *S.* Typhi or *S.* Paratyphi A at day 8, development of complications and relapse (re-occurrence of symptoms within 31 days after the start of treatment, both culture positive and negative). Using these definitions, the number of treatment failures between the ofloxacin and gatifloxacin group were similar (Table 2, Tables S1 and S2).

However, there was a statistically significant difference in FCT, defined as secondary endpoint, in favour of gatifloxacin in all three analysed populations, the patients infected with nalidixic acid-resistant isolates (median FCT, 4.70 days *versus* 3.31 days, Table 2), the culture confirmed population (3.99 days *versus* 3.30 days, Table S1) and also in the ITT population (2.15 days *versus* 1.97 days, Table S2). Indeed, in the predefined modified analysis, in which persistent fever on day 7 replaced persistent fever on day 10 in the composite endpoint, there was a significant difference in favour of gatifloxacin in all three groups. This highlights that the conclusions derived from such studies critically depend on the definitions chosen in the design of a clinical trial. At the present time there is no standardisation for the design of clinical trials in enteric fever. Hence in this study, the main difference between ofloxacin and gatifloxacin was the speed of resolution of fever, by ten days there was no difference. The slower resolution of fever in patients infected with nalidixic acid-resistant isolates during treatment with ofloxacin is corroborated by two previous studies [24], [21].

A trial conducted in adult patients in Vietnam between 1997 and 1998 [24], at which time the proportion of nalidixic acid-resistant strains increased from 10% to 76% (6), used a lower dose at 200 mg ofloxacin twice a day (an estimated 8 mg/kg/day) for a shorter duration of 5 days. Forty-four patients received ofloxacin, 53% were infected with nalidixic acid-resistant strains. The mean fever clearance time was 5.6 days in all 44 patients recruited, but it was prolonged to 7.25 days in the 21 patients infected with nalidixic acid-resistant isolates (60). Four out of 21 (19%) patients infected with nalidixic acid-resistant strains failed in the ofloxacin group. Three patients had “clinical treatment failure”, defined as the persistence of fever and symptoms for more than 5 days after the end of treatment (i.e. fever on day 10) and one patient relapsed. In 41% of patients, a transient stool carriage immediately after treatment was present. Another trial conducted from 1998 to 2001 in Vietnam used ofloxacin at 20 mg/kg/day for seven days (the same dose as used in this trial), and reported a “clinical treatment failure” rate of 36% (23 out of 63 patients) using the definitions “persistence of fever and at least one more symptom for more than 7 days after the start of treatment or the development of severe complications during treatment requiring a change in therapy” [21]. Ninety-eight percent of the isolates were nalidixic acid-resistant. All of the patients who failed had persistent fever and symptoms and 14 of those patients required retreatment. The mean FCT for patients treated with ofloxacin was 8.2 days. There was a high rate of faecal carriage immediately after treatment of 19% (12/62), potentially allowing transmission of isolates to close contacts and family members. The results of this study [21] are comparable to our data in the patients infected with nalidixic acid-resistant isolates, with the predefined modified analysis. When we applied the seven-day cut off for FCT, there were 21 out of 83 (25%) patients with treatment failure in the ofloxacin group, with 16 out of 83 (19%) patients still febrile on day 7. The reason for these slightly better results of ofloxacin in the enteric fever patients infected with nalidixic acid-resistant isolates in our trial could be that the tablets were weighed and pre-packed for each individual patient, whilst in the Vietnam studies the dose was estimated and patients may have received doses lower than the planned 20 mg/kg/day.

While all ofloxacin failures occurred in isolates with ofloxacin MIC>−0.125 mg/ml, 2 of the cases of gatifloxacin failure occurred even in MIC<0.19 mg/ml. One reason may be different gatifloxacin pharmacokinetics in these two patients.

These trials [24], [21] with a high proportion of nalidixic acid resistant-strains were included in a meta-analysis that analysed 7 trials (540 patients) that used ofloxacin for treatment [11]. There was a clear relationship between elevated ofloxacin MIC (MIC≥0.25 µg/mL) and prolonged fever clearance time and higher risk of treatment failure. This is corroborated by our data, Table 4 shows that patients infected with isolates with higher ofloxacin MICs (between 0.25 µg/mL and 0.75 µg/mL) [30] had longer median fever clearance times when treated with ofloxacin (median 4.76 days) than with gatifloxacin (median 3.31 days; p = 0.004). Crucially, patients and their guardians consider the fever to be the major symptom associated with enteric fever and clearly a prompt resolution of fever is an important issue in favour of gatifloxacin. We have data from our patients about their subjective perception of being cured from enteric fever and the majority of patients correlate this with the time the fever has subsided (A. Arjyal, manuscript in preparation). In addition, prolonged fever clearance times have been associated with microbiological failure, increased complication and relapse rates when using ciprofloxacin or ofloxacin for the treatment of enteric fever [31], [32].

Our study has a number of limitations. First, it was an open labelled randomized trial. Also, patients with severe enteric fever were not included in the study. Although faecal carriage rates were low in our study, in previous studies [21],[24], faecal carriage immediately after successful treatment of typhoid fever with ofloxacin was high and this may be a worrisome aspect of ofloxacin use. The lower fecal carriage in our population may be due to earlier presentation when stool tests are less likely to be positive or it may also be due to the intermittent nature of salmonella excretion in the stool. Notwithstanding these limitations, the findings of this study are of practical importance in many resource poor countries where enteric fever is endemic, nalidixic acid-resistant strains are common, and where ofloxacin is a standard drug for treatment of enteric fever [3]. Our patient population comprised of outpatients with uncomplicated enteric fever which reflects the situation of the majority of enteric fever patients receiving treatment in endemic countries. Our study also describes the ITT population which includes blood culture negative patients and shows that the results were consistent with the outcome in the blood culture-confirmed population. This is an important issue because undifferentiated fever of more than 3 to 4 days is treated empirically in most settings. A previous study from our hospital revealed that besides enteric fever, leptospirosis, and rickettsial (murine and scrub typhus) illnesses are other causes of such undifferentiated fever [1]. This present study would suggest that adequately-dosed gatifloxacin or ofloxacin would be an effective drug to empirically treat undifferentiated fever in our setting

Ofloxacin at a dose of 20 mg/kg/day remains an option to treat enteric fever, even in settings with high rates of nalidixic acid-resistance but leads to a slower resolution of symptoms compared to gatifloxacin. The convenience of once daily dosing of gatifloxacin and faster resolution of symptoms would suggest that gatifloxacin has advantages compared to ofloxacin for the treatment of young otherwise healthy patients with enteric fever in areas of nalidixic-acid-resistance.

## Supporting Information

Checklist S1 Consort checklist.
(DOCX)

Table S1 Summary of primary and secondary endpoints for patients with blood culture confirmed enteric fever.
(DOCX)

Table S2 Summary of primary and secondary endpoints for intention to treat population.
(DOCX)

Table S3 Incidence of adverse events in the intention to treat population during 15 days of follow up.
(DOCX)

## References

[1] Murdoch DR, Woods CW, Zimmerman MD, Dull PM, Belbase RH, et al (2004) The etiology of febrile illness in adults presenting to Patan hospital in Kathmandu, Nepal. Am J Trop Med Hyg 70: 670–675. 15211012

[2] Ochiai RL, Acosta CJ, Danovaro-Holliday MC, Baiqing D, Bhattacharya SK, et al (2008) A study of typhoid fever in five Asian countries: disease burden and implications for controls. Bull World Health Organ 86: 260–268. 18438514 10.2471/BLT.06.039818 PMC2647431

[3] Crump JA, Mintz ED (2010) Global trends in typhoid and paratyphoid Fever. Clin Infect Dis 50: 241–246. 20014951 10.1086/649541 PMC2798017

[4] Basnyat B, Maskey AP, Zimmerman MD, Murdoch DR (2005) Enteric (typhoid) fever in travelers. Clin Infect Dis 41: 1467–1472. 16231259 10.1086/497136

[5] Basnyat B (2010) Typhoid fever in the United States and antibiotic choice. JAMA 34 author reply 34–35. 20051567 10.1001/jama.2009.1935

[6] Parry CM, Hien TT, Dougan G, White NJ, Farrar JJ (2002) Typhoid fever. N Engl J Med 347: 1770–1782. 12456854 10.1056/NEJMra020201

[7] WHO (2003) The diagnosis, treatment and prevention of typhoid fever. Communicable Disease Surveillance and Response Vaccine and Biologicals 7–18 Available: http://whqlibdoc.who.int/hq/2003/WHO_V&B_2003.2007.pdf.

[8] Chau TT, Campbell JI, Galindo CM, Van Minh Hoang N, Diep TS, et al (2007) Antimicrobial drug resistance of Salmonella enterica serovar typhi in asia and molecular mechanism of reduced susceptibility to the fluoroquinolones. Antimicrob Agents Chemother 51: 4315–4323. 17908946 10.1128/AAC.00294-07 PMC2167998

[9] Chuang CH, Su LH, Perera J, Carlos C, Tan BH, et al (2009) Surveillance of antimicrobial resistance of Salmonella enterica serotype Typhi in seven Asian countries. Epidemiol Infect 137: 266–269. 18474127 10.1017/S0950268808000745

[10] Kariuki S, Revathi G, Kiiru J, Mengo DM, Mwituria J, et al (2010) Typhoid in Kenya is associated with a dominant multidrug-resistant Salmonella enterica serovar Typhi haplotype that is also widespread in Southeast Asia. J Clin Microbiol 48: 2171–2176. 20392916 10.1128/JCM.01983-09 PMC2884483

[11] Parry CM, Vinh H, Chinh NT, Wain J, Campbell JI, et al (2011) The influence of reduced susceptibility to fluoroquinolones in Salmonella enterica serovar Typhi on the clinical response to ofloxacin therapy. PLoS Negl Trop Dis 5: e1163. 21713025 10.1371/journal.pntd.0001163 PMC3119645

[12] Roumagnac P, Weill FX, Dolecek C, Baker S, Brisse S, et al (2006) Evolutionary history of Salmonella typhi. Science 314: 1301–1304. 17124322 10.1126/science.1134933 PMC2652035

[13] Renuka K, Kapil A, Kabra SK, Wig N, Das BK, et al (2004) Reduced susceptibility to ciprofloxacin and gyra gene mutation in North Indian strains of Salmonella enterica serotype Typhi and serotype Paratyphi A. Microb Drug Resist 10: 146–153. 15256030 10.1089/1076629041310028

[14] Parry CM, Beeching NJ (2009) Treatment of enteric fever. BMJ 338: b1159. 19493937 10.1136/bmj.b1159

[15] Effa EE, Lassi ZS, Critchley JA, Garner P, Sinclair D, et al (2011) Fluoroquinolones for treating typhoid and paratyphoid fever (enteric fever). Cochrane Database Syst Rev CD004530. 21975746 10.1002/14651858.CD004530.pub4 PMC6532575

[16] Thaver D, Zaidi AK, Critchley J, Azmatullah A, Madni SA, et al (2009) A comparison of fluoroquinolones versus other antibiotics for treating enteric fever: meta-analysis. BMJ 338: b1865. 19493939 10.1136/bmj.b1865 PMC2690620

[17] Lu T, Zhao X, Drlica K (1999) Gatifloxacin activity against quinolone-resistant gyrase: allele-specific enhancement of bacteriostatic and bactericidal activities by the C-8-methoxy group. Antimicrob Agents Chemother 43: 2969–2974. 10582891 10.1128/aac.43.12.2969 PMC89596

[18] Arjyal A, Basnyat B, Koirala S, Karkey A, Dongol S, et al (2011) Gatifloxacin versus chloramphenicol for uncomplicated enteric fever: an open-label, randomised, controlled trial. Lancet Infect Dis 11: 445–454. 21531174 10.1016/S1473-3099(11)70089-5 PMC3108101

[19] Dolecek C, Tran TP, Nguyen NR, Le TP, Ha V, et al (2008) A multi-center randomised controlled trial of gatifloxacin versus azithromycin for the treatment of uncomplicated typhoid fever in children and adults in Vietnam. PLoS One 3: e2188. 18493312 10.1371/journal.pone.0002188 PMC2374894

[20] Pandit A, Arjyal A, Day JN, Paudyal B, Dangol S, et al (2007) An open randomized comparison of gatifloxacin versus cefixime for the treatment of uncomplicated enteric fever. PLoS One 2: e542. 17593957 10.1371/journal.pone.0000542 PMC1891439

[21] Parry CM, Ho VA, Phuong le T, Bay PV, Lanh MN, et al (2007) Randomized controlled comparison of ofloxacin, azithromycin, and an ofloxacin-azithromycin combination for treatment of multidrug-resistant and nalidixic acid-resistant typhoid fever. Antimicrob Agents Chemother 51: 819–825. 17145784 10.1128/AAC.00447-06 PMC1803150

[22] Kalbfleish JD PR (2002) The statistical analysis of failure time data. Hoboken, NJ.: John Wiley and Sons.

[23] R Development Core Team. R: A language and environment for statistical computing. R Foundation for Statistical Computing,Vienna, Austria, 2012.

[24] Chinh NT, Parry CM, Ly NT, Ha HD, Thong MX, et al (2000) A randomized controlled comparison of azithromycin and ofloxacin for treatment of multidrug-resistant or nalidixic acid-resistant enteric fever. Antimicrob Agents Chemother 44: 1855–1859. 10858343 10.1128/aac.44.7.1855-1859.2000 PMC89974

[25] Park-Wyllie LY, Juurlink DN, Kopp A, Shah BR, Stukel TA, et al (2006) Outpatient gatifloxacin therapy and dysglycemia in older adults. N Engl J Med 354: 1352–1361. 16510739 10.1056/NEJMoa055191

[26] Ambrose PG, Bhavnani SM, Cirincione BB, Piedmonte M, Grasela TH (2003) Gatifloxacin and the elderly: pharmacokinetic-pharmacodynamic rationale for a potential age-related dose reduction. J Antimicrob Chemother 52: 435–440. 12917247 10.1093/jac/dkg370

[27] Vinh H, Anh VT, Anh ND, Campbell JI, Hoang NV, et al (2011) A multi-center randomized trial to assess the efficacy of gatifloxacin versus ciprofloxacin for the treatment of shigellosis in Vietnamese children. PLoS Negl Trop Dis 5: e1264. 21829747 10.1371/journal.pntd.0001264 PMC3149021

[28] Thwaites GE, Bhavnani SM, Chau TT, Hammel JP, Torok ME, et al (2011) Randomized pharmacokinetic and pharmacodynamic comparison of fluoroquinolones for tuberculous meningitis. Antimicrob Agents Chemother 55: 3244–3253. 21502621 10.1128/AAC.00064-11 PMC3122453

[29] Recherche pour le Developpement (2005): A Controlled Trial of a 4-Month Quinolone-Containing Regimen for the Treatment of Pulmonary Tuberculosis. Available: http://clinicaltrials.gov/ct2/show/record/NCT00216385.

[30] Parry CM, Thuy CT, Dongol S, Karkey A, Vinh H, et al (2010) Suitable disk antimicrobial susceptibility breakpoints defining Salmonella enterica serovar Typhi isolates with reduced susceptibility to fluoroquinolones. Antimicrob Agents Chemother 54: 5201–5208. 20837759 10.1128/AAC.00963-10 PMC2981260

[31] Rupali P, Abraham OC, Jesudason MV, John TJ, Zachariah A, et al (2004) Treatment failure in typhoid fever with ciprofloxacin susceptible Salmonella enterica serotype Typhi. Diagnostic microbiology and infectious disease. 2004 49 1: 1–3. 10.1016/j.diagmicrobio.2003.12.002 15135492

[32] Walia M, Gaind R, Mehta R, Paul P, Aggarwal P, et al (2005) Current perspectives of enteric fever: a hospital-based study from India. Annals of tropical paediatrics. 2005 25 3: 161–74. 10.1179/146532805X58085 16156980

