# Supplementary material for: Gatifloxacin Versus Ofloxacin for the Treatment of Uncomplicated Enteric Fever in Nepal: An Open-Label, Randomized, Controlled Trial
Source: PLoS Negl Trop Dis. 2013 Oct 31;7(10):e2523. doi: 10.1371/journal.pntd.0002523 (PMC3837022; doi:10.1371/journal.pntd.0002523)
Supplement: Table S1 — Summary of primary and secondary endpoints for patients with blood culture confirmed enteric fever. (DOCX) [file pntd.0002523.s002.docx]

|  | **Ofloxacin group**  **(n=109)** | **Gatifloxacin**  **(n=109)** | **Comparison** |
| --- | --- | --- | --- |
| **Time to treatment failure#**  Total number of pt with failures^$^  - Persistent fever on day 10  - Need for rescue treatment  - Microbiological failure  - Relapse until day 31  - Enteric fever-related   complications | 8  1  2  1  6  1 | 5  1  0  0  4  0 | HR=0.62 (CI 0.20 to 1.90), p=0.40 |
| **Risk of treatment failure*** | 0.08 (CI 0.02 to 0.13) | 0.05 (CI 0.01 to 0.09) | RD=-0.03 (CI -0.10 to 0.04), p=0.43 |
| **Median (IQR) time to fever clearance (days)*** | 3.99 (2.72 to 5.40) | 3.30 (CI 2.29 to 4.66) | HR=1.41 (CI 1.07 to 1.86), p=0.01 |
| **Relapses until day 31 - n**  - n blood culture-confirmed  - n syndromic  - Probability of relapse* | 6  4  2  0.06 (CI 0.01 to 0.11) | 4  2  2  0.04 (CI 0.001 to 0.08) | HR=0.66 (CI 0.19 to 2.35); p=0.52 |
| **Relapses until day 62 – n**  - n blood culture-confirmed  - n syndromic  - Proportion* | 11  5  6  0.11 (CI 0.05 to 0.18) | 8  3  5  0.08 (CI 0.03 to 0.14) | HR=0.72 (CI 0.29 to 1.79); p=0.48 |
| **Relapses after day 62 – n**  - n blood culture-confirmed  - n syndromic | 4  0  4 | 2  0  2 | - |

$ Patients may have more than one type of treatment failure.

* Kaplan-Meier estimates

HR=Hazard ratio (based on Cox regression), RD=absolute risk difference (based on Kaplan-Meier estimates), CI=95% confidence interval interval, IQR=inter-quartile range

n number of patients, pt patients

# Footnote: If persistent fever on day 7 (instead of day 10) would already be considered a treatment failure event (“modified analysis”), then there would be 23 treatment failures in the ofloxacin group *vs*. 12 in the gatifloxacin group (with 17 *vs*. 8 patients with persistent fever on day 7): HR=0.49 (CI 0.24-0.99), p=0.046.

**Supplementary table 1: Summary of primary and secondary endpoints for patients with blood culture confirmed enteric fever.**
