# Supplementary material for: Gatifloxacin Versus Ofloxacin for the Treatment of Uncomplicated Enteric Fever in Nepal: An Open-Label, Randomized, Controlled Trial
Source: PLoS Negl Trop Dis. 2013 Oct 31;7(10):e2523. doi: 10.1371/journal.pntd.0002523 (PMC3837022; doi:10.1371/journal.pntd.0002523)
Supplement: Table S2 — Summary of primary and secondary endpoints for intention to treat population. (DOCX) [file pntd.0002523.s003.docx]

|  | **Ofloxacin group**  **(n=316)** | **Gatifloxacin group**  **(n=311)** | **Comparison** |
| --- | --- | --- | --- |
| **Time to treatment failure^#^**  Total number of pt with failures^$^  - Persistent fever on day 10  - Need for rescue treatment  - Microbiological failure  - Relapse until day 31  - Enteric fever related   complications | 13  2  4  1  9  1 | 8  2  1  0  6  0 | HR=0.63 (CI 0.26 to 1.51), p=0.30 |
| **Risk of treatment failure*** | 0.04 (CI 0.02 to 0.07) | 0.03 (CI 0.01 to0.05) | RD=-0.02 (CI -0.05 to 0.01), p=0.30 |
| **Median (IQR) time to fever clearance (days)*** | 2.15 (0.90 to 4.27) | 1.97 (CI 0.89 to 3.67) | HR=1.20 (CI 1.02 to 1.42), p=0.03 |
| **Relapses until day 31 - n**  - n blood culture confirmed  - n syndromic  - Probability of relapse* | 9  6  3  0.03 (CI 0.01 to 0.05) | 6  3  3  0.02 (CI 0.004 to 0.04) | HR=0.68 (CI 0.24 to 1.91); p=0.46 |
| **Relapses until day 62 – n**  - n blood culture confirmed  - n syndromic  - Proportion* | 18  8  10  0.06 (CI 0.03 to 0.09) | 13  6  7  0.05 (CI 0.02 to 0.07) | HR=0.73 (CI 0.36 to 1.49); p=0.39 |
| **Relapses after day 62 – n**  - n blood culture confirmed  - n syndromic | 5  0  5 | 3  1  2 | - |

$ Patients may have more than one type of treatment failure.

* Kaplan-Meier estimates

HR=Hazard ratio (based on Cox regression), RD=absolute risk difference (based on Kaplan-Meier estimates), CI=95% confidence interval, IQR=inter-quartile range

n number of patients, pt patients

^#^ Footnote: If persistent fever on day 7 (instead of day 10) would already be considered a treatment failure event, then there would be 34 treatment failures in the ofloxacin group

*vs.* 19 in the gatifloxacin group (with 25 *vs.* 13 patients with persistent fever on day 7): HR=0.56 (CI 0.32-0.98), p=0.04.

**Supplementary table 2: Summary of primary and secondary endpoints for the intention to treat population**
