# Supplementary material for: Gatifloxacin Versus Ofloxacin for the Treatment of Uncomplicated Enteric Fever in Nepal: An Open-Label, Randomized, Controlled Trial
Source: PLoS Negl Trop Dis. 2013 Oct 31;7(10):e2523. doi: 10.1371/journal.pntd.0002523 (PMC3837022; doi:10.1371/journal.pntd.0002523)
Supplement: Table S3 — Incidence of adverse events in the intention to treat population during 15 days of follow up. (DOCX) [file pntd.0002523.s004.docx]

|  | **OFLOXACIN group (n=316)** |  | **GATIFLOXACIN group (n=311)** |  | **Comparison** |
| --- | --- | --- | --- | --- | --- |
| **TyT Type of adverse event event** | **number of patients** | **number of adverse events** | **number of patients** | **number of adverse events** | **p value** |
| Any selected AE | 215 (68.04%) | 415 | 223 (71.7%) | 421 | 0.33887 |
| abdominal discomfort | 0 (0%) | 0 | 1 (0.32%) | 1 | 0.49601 |
| abdominal pain | 67 (21.2%) | 67 | 60 (19.29%) | 60 | 0.61941 |
| anorexia | 51 (16.14%) | 51 | 47 (15.11%) | 47 | 0.74251 |
| arthralgia | 2 (0.63%) | 2 | 2 (0.64%) | 2 | 1 |
| back pain | 2 (0.63%) | 2 | 2 (0.64%) | 2 | 1 |
| burning micturition | 0 (0%) | 0 | 1 (0.32%) | 1 | 0.49601 |
| chest pain | 3 (0.95%) | 3 | 5 (1.61%) | 5 | 0.50191 |
| constipation | 1 (0.32%) | 1 | 1 (0.32%) | 1 | 1 |
| cough | 47 (14.87%) | 47 | 38 (12.22%) | 38 | 0.35223 |
| diarrhoea | 12 (3.8%) | 12 | 12 (3.86%) | 12 | 1 |
| dizziness | 44 (13.92%) | 44 | 51 (16.4%) | 51 | 0.43585 |
| dry mouth | 0 (0%) | 0 | 1 (0.32%) | 1 | 0.49601 |
| generalised discomfort | 0 (0%) | 0 | 1 (0.32%) | 1 | 0.49601 |
| headache | 15 (4.75%) | 15 | 21 (6.75%) | 21 | 0.30652 |
| hyperglycaemia | 0 (0%) | 0 | 1 (0.32%) | 1 | 0.49601 |
| insomnia | 6 (1.9%) | 6 | 0 (0%) | 0 | 0.03053 |
| joint pain | 0 (0%) | 0 | 1 (0.32%) | 1 | 0.49601 |
| myalgia | 14 (4.43%) | 14 | 10 (3.22%) | 10 | 0.53355 |
| nasal bleeding | 0 (0%) | 0 | 1 (0.32%) | 1 | 0.49601 |
| nausea | 76 (24.05%) | 76 | 77 (24.76%) | 77 | 0.85289 |
| palpitation | 0 (0%) | 0 | 1 (0.32%) | 1 | 0.49601 |
| rhinorrhoea | 1 (0.32%) | 1 | 0 (0%) | 0 | 1 |
| rigor | 2 (0.63%) | 2 | 3 (0.96%) | 3 | 0.68402 |
| skin rash | 4 (1.27%) | 4 | 3 (0.96%) | 3 | 1 |
| stomatitis | 1 (0.32%) | 1 | 3 (0.96%) | 3 | 0.36984 |
| sweating | 2 (0.63%) | 2 | 0 (0%) | 0 | 0.49923 |
| vomiting | 63 (19.94%) | 63 | 76 (24.44%) | 76 | 0.17975 |
| weakness | 2 (0.63%) | 2 | 2 (0.64%) | 2 | 1 |

AE adverse event

**Supplementary Table 3. Incidence of adverse events in the intention to treat population during 15 days of follow up**
